# Disgust as a primary emotional system and its clinical relevance

**DOI:** 10.3389/fpsyg.2024.1454774

**Published:** 2024-08-28

**Authors:** Alexey Tolchinsky, George F. R. Ellis, Michael Levin, Šárka Kaňková, Jeffrey S. Burgdorf

**Affiliations:** ^1^Professional Psychology Program, George Washington University, Washington, DC, United States; ^2^Department of Mathematics, University of Cape Town, Cape Town, South Africa; ^3^Allen Discovery Center at Tufts University, Wyss Institute for Biologically Inspired Engineering, Harvard University, Boston, MA, United States; ^4^Department of Philosophy and History of Science, Faculty of Science, Charles University, Prague, Czechia; ^5^Department of Biomedical Engineering, The Falk Center for Molecular Therapeutics, Northwestern University, Evanston, IL, United States

**Keywords:** disgust, affective neuroscience, psychopathology, OCD, immune system, active inference, psychotherapy

## Abstract

This paper advocates for considering disgust as a primary emotional system within Panksepp’s Affective Neuroscience framework, which has the potential to improve the efficacy of psychotherapy with obsessive-compulsive disorder, hypochondriasis, and emetophobia. In 2007, Toronchuk and Ellis provided comprehensive evidence that DISGUST system, as they defined it, matched all Panksepp’s criteria for a primary emotional system. A debate ensued and was not unambiguously resolved. This paper is an attempt to resume this discussion and supplement it with the data that accumulated since then on DISGUST’s relationship with the immune system and the role of DISGUST dysregulation in psychopathology. We hope that renewed research interest in DISGUST has the potential to improve clinical efficacy with hard-to-treat conditions.

## Introduction

In a review of the literature on disgust in the past 20 years, Knowles et al. (2018) mentioned that the scientific interest in disgust evolved from nearly non-existent when Phillips et al. (1998) declared it a “forgotten emotion in psychiatry” to 120 peer-reviewed papers that accumulated by 2018. We hope to contribute to this trend by discussing disgust’s broader definition as a full-featured, flexible emotional system closely related to the immune system. We think that this approach would allow clinicians to better identify disgust dysregulation in various kinds of psychopathology and treat it with sufficient priority, which may help improve the efficacy of psychotherapy with hard-to-treat clinical conditions.

An example of such a condition is a subtype of obsessive-compulsive disorder (OCD) with contamination obsessions and washing compulsions that has an acute onset following exposure to a traumatic event (Dykshoorn, 2014; Valderrama et al., 2020).^1^ The patients suffering from this condition present as having post-traumatic stress disorder (PTSD) symptoms and OCD symptoms concurrently, which significantly increases treatment complexity. Most patients diagnosed with any kind of OCD continue to experience substantial residual symptoms after successful initial treatment; at least a quarter of OCD patients do not improve with any treatments (Bloch and Pittenger, 2010). A third of the patients with acute PTSD do not reach stable improvement with any kind of treatment (Green, 2013). We believe that attending to disgust dysregulation early on in treatment is essential for cases of post-traumatic OCD with contamination obsessions. Similarly, we think that attending to disgust functioning early on is important in treating fear of vomiting (emetophobia) and illness anxiety (hypochondriasis).

We think that one of the reasons why disgust received less attention in psychiatry and clinical psychology compared to fear or anger was the viewpoint of some researchers who understood disgust as oral distaste. Therefore, prior to discussing disgust in detail, we find it useful to define the concepts related to disgust but distinct from it, such as distaste, nausea, retching, and vomiting. Toronchuk and Ellis (2007a) referred to distaste as animal’s oral rejection of bad tasting food. As an illustration, they mentioned an example from Kiefer and Orr (1992) of decorticate rats preferring sucrose and rejecting quinine.

Zhong et al. (2021) defined nausea, vomiting, and retching as follows:

Nausea is the unpleasant sensation of having the urge to vomit, whereas vomiting (emesis) is a physical event and is the forceful expulsion of intestinal and gastric contents through the mouth (Gelberg, 2018). Vomiting is often preceded by retching, where the content of gastrointestinal tract is forced into the esophagus, without expulsion of the vomitus (Gelberg, 2018). (p.1)

Toronchuk and Ellis (2007a) introduced the DISGUST^2^ system, which “arose phylogenetically in response to danger to the internal milieu from pathogens and their toxic products (p. 1799).” They also suggested that DISGUST had the capacity for the anticipatory protection of the internal milieu. This capacity is thought to be achieved by flexibly using a variety of actions in anticipation of an exposure to a potentially noxious material prior to the organism’s contact with such material (p. 1800).

Toronchuk and Ellis (2007a) highlighted the difference between the organisms’ defense from external threats (e.g., predators), which was the function of Panksepp’s (1998) FEAR system, and its defense from noxious microorganisms that had the potential to get into the body, which was the function of DISGUST. They further inferred that there was a relationship between the organism’s immune system and DISGUST and that DISGUST was likely phylogenetically older than FEAR.

To summarize, Toronchuk and Ellis (2007a) defined DISGUST as a flexible emotional system that was longer lasting than distaste and not constrained to the gustatory aversive reaction; it could be activated by multiple pathways, including olfactory, visual, or other sensory stimuli, and by the higher cognitive processes; it had a function of protecting the organism’s internal milieu from pathogens and their toxic products by enabling a variety of emotional responses in advance of the organism’s contact with potentially noxious material.

As you can see the DISGUST system as defined by Toronchuk and Ellis (2007a) is qualitatively different from distaste, which is indeed a relatively inflexible, low-level, short-lived phenomenon rather than a full-featured, flexible emotional system. To illustrate the difference, in the above example with decorticate rats, the functioning of the low levels of the brain stem were sufficient for rats to prefer sucrose and reject quinine, while the functioning of DISGUST requires subneocortical processing, including the insula (Toronchuk and Ellis, 2007a).

Accordingly, the capacity for DISGUT for launching anticipatory protective actions in a flexible manner, suggests the use of memory systems capable of recording more context and a wider set of choices than those involved in a simple reflex. We will describe the recent research on such memory systems in the “Neuroanatomical substrates of DISGUST” section. As an illustration of a wider range of reactions, in humans, we not only avoid the foul-smelling items, but we also have a characteristic facial expression (Ekman and Friesen, 1986), which is conceptualized at least in part as a social signaling mechanisms to others (Toronchuk and Ellis, 2007a). To illustrate multiple pathways leading to the activation of DISGUST in humans, we think that in addition to taste, smell, and vision, Miller’s (1997) example is descriptively rich in reference to tactile associations with DISGUST: “slimy, slithery, viscous, oozing, festering, scabby, sticky, and moist” (p. 19).^3^

An additional point of debate is an attempt by some theorists to limit DISGUST to nausea, retching or vomiting. We would like to highlight our view that nausea, retching, or vomiting are closely related to DISGUST in function (Zhong et al., 2021) but are not identical to it in all circumstances^4^ (Fessler et al., 2005; Kaňková et al., 2022; Kaňková et al., 2023a; Kaňková et al., 2023b; Fejzo et al., 2023). Specifically, a sensation of having an urge to vomit is not a necessary component of all DISGUST processes. For example, a tactile sensation of “slimy” may activate DISGUST, without any food ingestion or nausea. Retching and vomiting are relatively inflexible reflexive reactions to noxious material already detected in the organism, while DISGUST has the capacity for anticipatory protection. We see oral distaste, nausea, retching, and vomiting as only a subset of the possible processes related to DISGUST but not identical to it. We summarize the differences in Table 1 below.

**Table 1:** Differences between nausea, vomiting, retching, and DISGUST.

	Definition	Features
Nausea	An unpleasant sensation of having the urge to vomit	Affect, related to vomiting
Vomiting	A physical event—the forceful expulsion of intestinal and gastric contents through the mouth	Action, relatively inflexible
Retching	A process where the content of gastrointestinal tract is forced into the esophagus, without expulsion of the vomitus	Action, relatively inflexible
DISGUST	A flexible emotional system that is longer lasting than distaste and not constrained to the gustatory aversive reaction; it can be activated by multiple pathways, including olfactory, visual, or other sensory stimuli, and by the higher cognitive processes; it has a function of protecting the organism’s internal milieu from pathogens and their toxic products by enabling a variety of emotional responses in advance of the organism’s contact with potentially noxious material.	A complex emotional system capable of anticipatory protection of the organism’s internal milieu. It contains an affective component—the feeling of being disgusted, which is qualitatively different from nausea; it has flexible and multi-sensory perceptual components, memory, connections to the higher cognitive processes, and diverse action components.

The definitions of vomiting, retching, nausea are from Zhong et al. (2021).

In terms of the homeostatic regulation of DISGUST system, the homeostatic settling point (Solms, 2018b) is a state where the internal milieu is perceived as being safe from pathogens. A negatively valenced subjective affect of DISGUST indicates that the internal milieu is in danger of infection, the higher the threat and the stronger the feeling. When the system is taking actions to minimize this threat, the valence becomes positive, as the system is on its way of returning to the settling point, and when the settling point is reached, the feeling is no longer present, which is the contentment phase of DISGUST.^5^

As a clinical illustration, we suggest that a feeling indicating a chronically unmet need (Solms, 2018a) for the internal milieu to be safe from pathogens is one of the features of OCD with contamination obsessions and washing compulsions. We would like to add this consideration to one of the earlier models described by Jackson and Solms (2013) who suggested that OCD was accompanied by dysregulated PANIC (Panksepp and Biven, 2012). We think that DISGUST and PANIC can be dysregulated concurrently, and that DISGUST dysregulation should not be reduced to FEAR or PANIC dysregulation, since the protection of the internal milieu is a qualitatively different affect than the feeling of fear (e.g., of predators) or a feeling of separation distress.

This broader framework of DIGUST allows us to infer a possible evolutionary progression from a simple inborn reflex of distaste (e.g., preference sucrose and rejection of quinine) to a more flexible system of distaste augmented by learning to activate retching and vomiting when specific pathogens are detected inside the body, accompanied by a feeling of nausea, which facilitates learning to avoid the stimuli associated with specific pathogens (Xie et al., 2022). Then, in the next phase of evolutionary development, we can see the previously described systems augmented by the most flexible, context-dependent, longer-lasting system of DISGUST, working in concert with the immune system, which allows sophisticated anticipatory and defensive strategies and the highest level of adaptation to the environment. For example, humans can be informed of possible pathogens long before they are exposed to contact with these pathogens when seeing a facial expression of disgust in others or watching news on TV about the COVID-19 pandemic.

Based on the significantly broader scope, Toronchuk and Ellis (2007a) raised a possibility of DISGUST being considered a basic emotional system^6^ on par with Panksepp (1998) SEEKING, FEAR, RAGE, LUST, PLAY, CARE, and PANIC.

Panksepp (2007) outlined the following criteria for basic emotional systems:

(1) They should be accessed by certain unconditional environmental stimuli. (2) They should generate a coherent set of behavioral actions and supportive physiological responses. (3) They should be able to gate inputs from the environment. (4) They should be capable of sustaining emotive activity for a substantial period after the precipitating events have passed. (5) Emotional responses should be capable of being triggered by cognitive activities. (6) Emotions should be capable of activating and regulating complex cognitive strategies. And (7) psychiatrically relevant affective experience must be generated by such brain systems. (p. 1820, see text footnote 1)

Toronchuk and Ellis (2007a,b) provided comprehensive review of anatomical, functional, phylogenetic, and behavioral data in support of DISGUST meeting the first six criteria. We will not repeat all the points they made at length in this paper. As a summary, they provided data that (a) DISGUST relied upon the distinct neural circuitry (reviewed in the below section “Neuroanatomical substrates of DISGUST”), which could be unconditionally accessed by various sensory stimuli and not just one pathway; (b) that DISGUST system could organize diverse behaviors instead of being a relatively simple reflex (e.g., DISGUST activation leads to the characteristic facial expression in humans, avoidance of conditionally learned stimuli, augmentation of the immune system functioning when it is downregulated, etc.); (c) change hedonic value in taste or other senses (which simple reflex of distaste cannot accomplish); (d) these hedonic changes could outlast the immediate exposure to a stimulus; (e) and that DISGUST was activating and regulating complex cognitive activities, (e.g., the blending of primary DISGUST with complex cognitions leading to moral disgust).

In Toronchuk and Ellis (2007b) response to Panksepp (2007) critique of their first paper, they subsequently provided data in support of Pansepp’s seventh criterion: “psychiatrically relevant affective experience must be generated by such brain systems.” We think, however, that Panksepp’s seventh criterion is a matter of opinion and cannot be independently verified. Unlike malaria or flu, we do not have clear, unambiguous, widely agreed-upon etiological explanations for most psychiatric conditions; we have conflicting opinions of various theorists, accompanied by ongoing debates. Using the seventh criterion to define a basic emotion may therefore be an example of a circular logic. Despite such complexity, we will attempt in this paper to provide additional data in support of DISGUST’s relevance for thinking about psychopathology and clinical work.

We consider this paper in combination with Toronchuk and Ellis (2007a,b) work as an attempt to renew research interest in DISGUST as a primary emotional system. We realize that more empirical data are necessary and hope that this series of papers presents a roadmap for future research, including the experimental studies in animals and humans, as well as further investigation of the underlying neuroanatomy of DISGUST and its dynamics. While this research is ongoing, our hope is to provide the framework for necessary adjustments in the clinical models and clinical practice, which have been negatively affected by viewing disgust as merely distaste or nausea.

In what follows, we will describe the evolutional origins of protecting the internal milieu of any organism. We will then discuss the literature that emerged since 2007 on the complex relationship between DISGUST and the immune system. Subsequently, we will review the neuroanatomical substrates of DISGUST. Then, we will introduce additional terms for emotional concepts relevant to the clinical practice and, finally, describe the literature on the diagnosis and treatment of clinical conditions, where we think DISGUST plays a major role.

## Some constraints in the research methodology of affective neuroscience

One of the primary experimental methodologies Panksepp and colleagues used to map out the neuroaffective systems was deep brain stimulation (DBS) in rats (Panksepp, 1982). Rats are non-emetic species—they do not retch or vomit, making it practically difficult to come up with observable signs corresponding to their reactions to pathogens in their internal milieu or the environment. We think that this methodological limitation was the practical constraint of investigating disgust in rats or mice for decades, resulting in the scarcity of the research literature on disgust in non-emetic species.

To our knowledge, the first rigorous research methodology of investigating defensive reactions similar to but not identical with nausea, retching, and vomiting in non-emetic species, was developed by Xie et al. (2022). Not only they described and tested the specific behavioral signs corresponding to retching-like behavior in laboratory mice, but they also used a genetic labeling of specific neurons with subsequent chemogenetic inactivation of them, which is a more granular technique than DBS. These newer methods relying on the Translating Ribosome Affinity Purification (TRAP) methodology were not available to Panksepp and his colleagues in the 1980s and 1990s, when the bulk of experimental studies underlying Panksepp’s affective neuroscience were conducted.

## The ancient process of defining and determining the internal milieu

Most discussions of physiology, as well as psychology and philosophy of mind, take for granted the existence of a self (typically, a modern adult human) as the subject of various drives, the owner of goals and memories, and the bedrock with respect to which we can try to understand behavior and inner experience. But, in seeking the origins of fundamental aspects of mind, it is critical to acknowledge that all intelligence is collective intelligence: we are made of parts (cells), which were once individual organisms themselves, and we all began life as a single cell – a quiescent oocyte (Levin, 2019). Each of us personally took the remarkable journey from simple matter to complex mind, not just on the evolutionary timescale but also on the deeply individual timescale of embryogenesis (Levin, 2022). This aspect of metazoan biology emphasizes autopoiesis (self-construction, of body and mind) (Maturana and Varela, 1980; Varela et al., 1974) as a possible context and origin of fundamental aspects of cognition.

Imagine an amniote blastoderm, with many thousands of cells. Each cell is some other cell’s “external world.” The traditional view is that this is one “embryo,” which will give rise to one “self” (e.g., a human being). But, introducing temporary scratches into this blastoderm will result in islands of self-organization, each of which will form a separate embryo (Lutz, 1949). If they heal together, the result is conjoined twins. Thus, a single blastoderm is a pool of somatic and cognitive potentiality, from which may emerge 0, 1, or some number of individual Selves. This process reveals that the construction of a Self, and its boundary (physical, physiological, and eventually, psychological), is not a hardwired, inevitable, genetically-determined process but an active example of autopoiesis during which the emergent system must autonomously determine where it ends and the “outside world” begins (Fields and Levin, 2020, 2023; Friston, 2013; Friston et al., 2015; Friston et al., 2020).

Internal models of the self/world boundary are as essential for body morphogenesis and physiological allostasis (McEwen, 1998; Schulkin and Sterling, 2019) as they are for subsequent functional behavior. The borders of the organism, the available sensors, and directly controllable effectors, are not pre-given but must be discovered dynamically (Bongard et al., 2006). Whether during the default (singleton) state, or during the more rare twinning events, each “embryo” must have a kind of competency to dynamically determine which cells are its own components, and which cells are outside world. Moreover, the constant threat of parasitisim and various kinds of biological hijacking of cellular competencies (Fields and Levin, 2022; Levin, 2023) exerts continuous adaptive pressure on biological agents to be good at distinguishing not only whether passive features of the environment (nearby cells) are part of them or not, but also at the second-order task of knowing whether various signaling stimuli (e.g., control signals) are being issued by “my own mechanisms” or some external agent that seeks to exploit. Thus, it is reasonable to expect that neural functions, which are thought to derive from the same bioelectrical computational mechanisms used to orchestrate the formation of the body (Fields et al., 2020), will retain this fundamental, ancient imperative: preserve the boundary between the Self and the outside world by detecting and rejecting matter, energy, information, and influence that seeks to infiltrate that boundary. This may be an origin of a primary sense of disgust, as recognition of a transgressed boundary by a “foreign” agent that is judged to be external and not belonging inside the somatic or psychological boundary of the Self.

In line with the notion that DISGUST can be seen as a primordial aspect of basal cognition, the close relationship between DISGUST and the immune system foregrounds the role of the immune system in self-organization in the face of environmental challenges, such as infection. One key challenge for the immune system is to disambiguate between the Self and the Nonself, invoking the notions such as Thymic Self (Geenen, 2021), and attendant theoretical considerations of what it means to represent the Self at any level (Markose, 2022). This issue is particularly relevant in situations where the distinction between the Self and the Nonself becomes catastrophically distorted—as in cancer^7^—or experiences changes—as in pregnancy. Pregnancy is a particularly interesting example, in which a partial, functional down-regulation of the mother’s immune response allows the maternal immune system to suspend its attack of the Nonself tissue—the fetus. We will return to pregnancy later and consider the evidence for a compensatory increase in sensitivity to disgust at systemic and conceptual levels.

## The evolutionary development of DISGUST

Defending against pathogens such as bacteria, viruses, protozoa, or helminths, which are ubiquitous in our environment, represents one of the most significant driving forces of evolution. DISGUST can be seen as a mammalian adaptation to a life threatened by parasites and microbes (Curtis et al., 2004). Evolutionarily, it can be seen as a very old defense system whose purpose was protection from disease.

Although many different evolutionary perspectives have emerged over time to explain the diverse adaptive functions related to disgust (e.g., Tybur et al., 2009), one of its functions may have been to serve as a mechanism for orally rejecting harmful substances (Rozin and Fallon, 1987). Rozin and Fallon (1987) attributed the role of disgust in evolution to the advantage of maintaining a clean nest environment, as moist and soft body excretions provided a suitable environment for the proliferation of microorganisms, and ingesting them could lead to the spread of disease.

In line with the protective function of disgust against pathogens, Haidt et al. (1994) suggested that disgust in humans was elicited by spoiled food, animals carrying infections, body excreta, or low hygiene standards. They also noted other circumstances where people could experience disgust, such as while looking at wounds, blood, or dead bodies.

As human society has evolved, psychological or cultural “extensions” of disgust developed, where stimuli that were not inherently related to the protection of the internal milieu from pathogens were described as “dirty,” or “clean,” such as “unclean thoughts” or certain kinds of sexual preferences as they are presented in religious texts. Chapman and Anderson (2013) referred to this phenomenon as “moral disgust” and expressed a viewpoint in agreement with Toronchuk and Ellis (2007a) that higher cognitive processes could elicit the biological state of disgust^8^.

A related but different phenomenon was a need to distance oneself from unwanted sexual contact initiated by others, leading to humans reporting feeling disgusted in such circumstances, in addition to being scared, helpless, or angry (Brake et al., 2021).

## Neuroanatomical substrates of DISGUST

Toronchuk and Ellis (2007a,b) described a complex network of brain regions participating collectively in DISGUST. They highlighted the anterior insular cortex (aIC) as a key region that was necessary but likely not sufficient for DISGUST functioning. They summarized numerous functional imaging studies in healthy human subjects, as well as intracerebral event-related potentials data recorded with depth electrodes implanted during presurgical evaluation of patients with treatment-resistant temporal lobe epilepsy. Additionally, they reviewed studies showing that lesions to aIC in humans disrupted both the experience of DISGUST and the recognition of DISGUST in others. More recent human functional imaging studies confirmed anterior insula as a key region involved in disgust processing (Jabbi et al., 2008; Pujol et al., 2018; Gan et al., 2022).

In animal research, conditioned taste aversion (CTA) has been used to study some processes related to disgust. CTA is an associative learning paradigm, closely associated with avoidance of odors or tastes associated with a possible illness. CTA in rats and mice depends on the aIC and the amygdala (see Toronchuk and Ellis, 2007a for a review; Tuerke et al., 2012; Kayyal et al., 2019). Several studies suggest more specifically that basolateral amygdala (BLA) is a necessary region involved in CTA functioning (Kayyal et al., 2019; Lavi et al., 2018; Inui et al., 2019).

Inui et al. (2019) suggested that BLA was involved in the retrieval of CTA-related memories in male rats. Kayyal et al. (2019) conducted experiments with mice and made a stronger claim that the activation of the neuronal projections from aIC to BLA was necessary and sufficient to form and retrieve aversive taste memory. Based on these data, we see the memory engrams encoded in a network including the aIC, BLA and the projections between then, as a more flexible, programmable memory system allowing a wider range of antecedents and responses than the memory systems underlying the brainstem-based reflex of distaste.

In addition to aIC and BLA, Toronchuk and Ellis (2007a) reviewed three studies by Sprengelmeyer et al. (1996, 1997, 2003) with human patients diagnosed with Parkinson’s, Huntington’s, obsessive-compulsive disorders, suggesting a possible role of basal ganglia (BG) in DISGUST. Chapman and Anderson (2012), review suggested mixed and inconclusive results with respect to BG’s involvement in disgust. One of the possible reasons for that is thought to be lateralization—two clinical case reports suggested that only left hemisphere lesions in the basal ganglia disrupted DISGUST (Calder et al., 2000; Straube et al., 2010). More recently, Holtmann et al. (2020) examined eight patients with unilateral damage to the insula – basal ganglia system (IC-BG system) and compared them to healthy controls. They also concluded that left-hemisphere damage to this system impaired disgust. Craig (2002) supported the lateralization of functions in the insula. To summarize, more data is needed to clarify the possible involvement of BG in DISGUST.

When describing one of the possible processes related to the DISGUST activation—reflexive vomiting, Toronchuk and Ellis (2007a,b) reviewed studies showing that in mammals, nucleus tractus solitarius (NTS) in the medulla was involved. In a more recent review, Zhong et al. (2021) expanded the list of the key regions mediating vomiting to the brainstem sites located in the dorsal vagal complex (DVC) of the medulla, including the area postrema (AP), the NTS, and the dorsal motor nucleus of the vagus (DMV). An important feature of AP and NTS is that they lack the blood–brain barrier, which allows the neurons located in these sites to respond quickly to the stimuli circulating in blood and cerebrospinal fluid (Zhong et al., 2021).

Neuroanatomy of vomiting or retching has not been tested in non-emetic species rigorously prior to 2022, when the results reported previously for mammals were essentially supported by Xie et al. (2022) experiments. Xie et al. (2022) replicated retching-like behaviors in mice that ingested toxin-contaminated food. Xie et al. (2022) used genetic labeling technique to show the abundant activation of neurons in the NTS, AP, and DMV. Subsequently, they used chemogenic deactivation of these neurons that impaired retching-like behaviors in mice that ingested toxins.

Toronchuk and Ellis (2007a) reviewed studies showing that while the aIC, the amygdala and the NTS were all involved in DISGUST processing, the taste, and interoception pathways that traverse these regions differed in rodents and primates. In primates, the amygdala appears to receive interoceptive input from the insula (Mesulam and Mufson, 1982; Evrard, 2019; Chen et al., 2021) and not via the subcortical connections from the NTS, as in rodents. The same can be said about the taste inputs to amygdala from the insula in primates (Rolls, 1989).^9^ In addition, the interoceptive pathway in primates and not rodents has a direct connection from the NTS to the thalamus, bypassing the parabrachial nuclei in the pons (Craig, 2002). Craig interpreted these differences as follows: “These functional anatomical considerations indicate clearly that primates differ from sub-primates in the encephalization of a direct cortical image of the physiological condition of the body (p. 663).”

The pathway differences between rodents and primates are not unique to DISGUST system and are observed in other primary neuroaffective systems mapped out by Panksepp et al. For example, dorsal striatum is involved in SEEKING system (Alcaro and Panksepp, 2011); a recent review by Lee et al. (2023) reported that five sensory inputs from the cortex and the thalamus converged on the tail of the striatum (TS) in rodents, but not in primates. The thalamus plays a role in PLAY system (Siviy and Panksepp, 1985) and PANIC system (Panksepp and Biven, 2012). Joyce et al. (2021) showed that in primates but not in rodents, the reuniens nucleus (RE) of the thalamus contained inhibitory neurons and has robust connections with the amygdala. In addition, Morgan and Amaral (2013) reported that primates and rodents have structural (and likely functional) differences in amygdala. We think that these differences can possibly affect RAGE and FEAR systems (Panksepp and Biven, 2012).

Lee et al. (2023) documented other important differences in primate and rodent brains, including developmental, morphological/anatomical, and functional sensory differences (e.g., higher development of visual connections in primates as compared to rodents that have relatively poor vision). These data suggest that caution is necessary when applying research in rodents to primates, including humans. Panksepp and Biven (2012) wrote extensively about this topic in Chapter 13 of their book.

An additional level of complexity in species-specific data is Craig’s (2002) suggestion that humans and not monkeys have possibly developed “sequential re-representations of the physiological state of the body in the right anterior insula (p. 663).” He further inferred that this lateralized re-representation in humans corresponded with bodily self-awareness.

In line with Panksepp’s model of the primary emotional systems, we see DISGUST as a multi-tiered hierarchy. There is an inborn reflex of distaste, which can function with brain-stem-based, relatively inflexible programs, without the involvement of subneocortical structures (Steiner et al., 2001, as cited in Toronchuk and Ellis, 2007a). Further up in the hierarchy, perhaps at the secondary level described by Panksepp, the components of the DISGUST network including the aIC, BLA, and possibly the basal ganglia, allow for more flexible and malleable memories involved in the CTA. At a third level of the hierarchy, we may see the most flexible and context-dependent neocortex-based learned memories, such as verbal associations. An example of a phenomenon at that level could be moral disgust, or verbal associations with creepy and oozing objects. As Panksepp and Biven (2012) suggested, there is a complex and dynamic set of top-down and bottom-up influences between the three layers, which makes the entire system dynamic and evolving in the process of development and adaptation.

Toronchuk and Ellis (2007a) illustrate this hierarchical structure by an example that lesions to human aIC disrupt both the experience of DISGUST and the recognition of DISGUST in others while innate distaste responses are found in decerebrate mammals, including anencephalic human infants (Steiner et al., 2001).

It is also useful to acknowledge, following Toronchuk and Ellis (2007a), that the insula activity occurs not exclusively during DISGUST activation but also in other emotional contexts. This observation applies to other neuroaffective systems as well, such as the amygdala and the thalamus playing a role in at least two primary neuroaffective systems. In part, this phenomenon of a single region implicated in multiple emotional systems is related to a clear simplification of naming a large and complex part of the brain such as the insula as a functionally meaningful and sufficient whole, decontextualized from the areas it is connected to (or even mentioning one of its parts, such as anterior insula). Indeed, the processing of taste, interoceptive and other data in primates is complex and a new, more fine-grain model of processing these signals has been proposed (Evrard, 2019). Additionally, a one-to-one anatomical to functional mapping is inadequate in the brain, nearly every brain region is involved in multiple functions and often a single function is performed by a network comprising multiple regions (Solms and Turnbull, 2018).

## DISGUST and the immune system

In support of Toronchuk and Ellis’ insight that the insula, as one of the contributing regions to the DISGUST system was possibly related to the immune system’s functioning,^10^ Koren et al. (2021) showed experimentally in mice that Insula Cortex (InsCtx) was used to both store and retrieve immune-related information. They induced colitis with dextran-sulfate-sodium (DSS) as a possible example of gastrointestinal inflammation. Then, they used activity-dependent cell labeling and compared the mice that were given DSS with controls.

Their results showed that induced colitis in mice was associated with increased activation of insula neurons. In the next phase, 4 weeks after recovery, they reactivated the specific InsCtx neuronal ensembles they observed in the previous experiment and detected heightened immune activity in the colon. Subsequently, they repeated these experiments with another condition—zymosan-induced peritonitis (ZIP), which is immunologically distinct from DSS-induced colitis; and they obtained similar results. They also showed that the neuronal ensembles in the insula specific to ZIP were distinct from those involved in DSS-induced colitis.

In the last two decades, many papers have focused on the relationship between DISGUST and the immune system. On the premise that DISGUST is the affective part of the so-called “behavioral immune system^11^” (Schaller and Duncan, 2007)—an important mechanism protecting individuals against pathogens—Fessler and Navarrete (2003) formulated the Compensatory Prophylaxis Hypothesis (CPH). This hypothesis stated that increased activity in the disgust system functioned to augment the protection of the internal milieu when the immune system was partially suppressed or downregulated.

The CPH was originally formulated in the context of changes in levels of progesterone and associated changes in immunosuppression during the menstrual cycle (Fessler and Navarrete, 2003) since progesterone was thought to have immunosuppressive effects (Miyaura and Iwata, 2002). Therefore, in the luteal phase, when progesterone is the highest, disgust sensitivity should also be increased to compensate for the immunosuppression.

While some studies supported this hypothesis of CPH’s relationship with progesterone levels, (Fleischman and Fessler, 2011; Milkowska et al., 2019; Olatunji et al., 2020; Miłkowska et al., 2021; Želaźniewicz et al., 2016), others found no correlation between disgust and progesterone levels or cycle phase (Fessler and Navarrete, 2003; Stern and Shiramizu, 2022; Jones et al., 2018; Timmers et al., 2018). In response to the study by Jones et al. (2018) and Fleischman and Fessler (2018) argued that disgust responses might have been upregulated to partially compensate for reproductive immunomodulation, but that progesterone either did not drive such changes or interacted with other responsible physiological components. Disgust may also be upregulated in relation to other forms of immunomodulation.

Stevenson et al. (2009) found that recently sick people have shown increased disgust sensitivity (a measure of how negatively individuals consider the experience of disgust, see Haidt et al., 1994), and Sarolidou et al. (2020) showed increased disgust sensitivity in people who considered themselves more vulnerable to disease. Moreover, recent studies showed higher disgust sensitivity in women with an infection in the luteal phase of the menstrual cycle (Milkowska et al., 2019) or in women who reported recent health problems in the first trimester of pregnancy (Dlouhá et al., 2023).

This period of pregnancy, which is accompanied by significant hormonal changes and immunomodulation, is another important area of CPH research. Two studies (Fessler et al., 2005; Żelaźniewicz and Pawłowski, 2015) have shown elevated disgust sensitivity in the first trimester, a time of many developmental processes sensitive to disruption when the mother and embryo need increased protection against pathogens.

The first direct evidence of immune system activity in association with disgust sensitivity in pregnancy was recently provided by Kaňková et al. (2022). The authors showed that elevated disgust in the first trimester was associated with decreased levels of certain cytokines, proposing that disgust compensated for insufficient immune adaptation in early pregnancy. Furthermore, Kaňková et al. (2023a) showed significant negative correlations between disgust sensitivity and free β-human chorionic gonadotropin (hCG) levels, the hormone contributing to establishing pregnancy-induced immune tolerance (Schumacher et al., 2013), in women in the first trimester of pregnancy. In a follow-up study, Kaňková et al. (2023b) reported the activation of the DISGUST system in pregnant women in response to the heightened environmental risk of infection (such as during the COVID-19 pandemic), was rather weak because disgust had already been elevated due to pregnancy.^12^

Thus, CPH serves as a coherent current model as to why people who need to be more protected from infectious diseases (such as more vulnerable periods related to women’s reproduction) experience a hyper-activation of DISGUST.^13^

## Why is DISGUST being a primary emotional system important to psychiatrists and psychotherapists?

Sasson et al. (2005) published a descriptive case series of 13 Israeli veterans with comorbid OCD and PTSD. The time of OCD onset corresponded with exposure to a traumatic event in all 13 cases. Moreover, specific OCD symptoms were associated with particular types of trauma. As one category of these OCD types, contamination obsessions and washing compulsions were prevalent among patients, who survived traumatic events where the feeling of intense disgust was present.

For example, Sasson et al. (2005) described a case of a patient who survived a terrorist attack, resulting in the patient’s body being covered with the flesh of the terrorist, and another case of the sexual abuse of a patient while he was in captivity. These patients developed contamination obsessions and washing compulsions. Similarly, traumatic events related to accidentally harming others were related to patients’ checking rituals. Thus, Sasson et al. (2005) inferred that specific obsessions and compulsions in posttraumatic OCD were related in content to the theme of the trauma. De Silva and Marks (1999) came to a similar conclusion; they described a case of a woman, who developed a contamination obsession and compulsive washing soon after being sexually assaulted.

We think that there is indeed a specific quality of symptoms in these cases, and it would be lost if post-traumatic OCD were explained as merely an anxiety disorder, basal ganglia disorder, or any other kind of overly reductive model. The various symptoms of these patients could be considered co-occurring phobias, anxiety disorders, mood disorders, sleep disorders, and interpersonal challenges. We think that these patients also suffered from dysregulated DISGUST.

We advocate in this paper for a “both-and” approach to these clinical cases, as compared to “either-or” models, which suggest that these issues can be reduced to a single factor.

For example, consider human reactions to spiders. Panksepp’s FEAR system is activated in such experiences; and recent studies suggest that arachnophobia could be innate (Hoehl et al., 2017). However, spiders are also associated with “hairy” or “creepy” concepts (Olatunji et al., 2017), which evoke another reaction—DISGUST and a threat of contamination or illness in addition to and not instead of the perception of spiders as external threats (Polák et al., 2020). The same can be said about snakes being perceived as not just scary, but also “slimy,” which evokes DISGUST.

Further, consider on the one hand, the abovementioned definition of DISGUST by Toronchuk and Ellis (2007a) as a system protecting the organism’s internal milieu from pathogens (p. 1799) and, on another hand, health-related anxiety or hypochondriasis. As Knowles et al. (2018) suggested, there is likely a relationship between disgust and health-related anxiety. We can add that such a relationship seems hardly accidental, considering that the activation of DISGUST, as Toronchuk and Ellis described it, is precisely supposed to elevate the threat of illness. Finally, consider the fear of vomiting (emetophobia). Van Overveld et al. (2008) described the evidence of a connection between this phobia and disgust sensitivity.

In what follows, we try to make a case that considering DISGUST in the understanding and treatment of these conditions from the get-go is essential. Before doing that, we need to discuss some additional conceptual factors pertinent to clinical practice.

## Additional terminological considerations

Adolphs (2017) defined emotions as “functional biological states” and he distinguished this term from (a) our conscious experiences of emotions, referred to as “feelings” (b) our ability to represent emotions and think about them—“emotional concepts” (c) our ability to talk about emotions with others—“semantic knowledge of emotions,” (d) behaviors caused by emotional states—“expressions of emotions” (e) ability to attribute emotions to others – “attribution.” One can consider the functional biological states and terms (a–e) as various categories of terms related to emotions. Adolphs discussed the complex relationships between them and the boundaries of each category and provided evidence of possible clinical dissociation between some of them.

One of the categories Adolphs defined that requires clarification is “emotional concepts.” As an illustration, when a baby integrates interoceptive and exteroceptive streams while experiencing low blood sugar levels, she establishes a higher-level domain-general representation (belief) of an emotional state, such as hunger. This is a level of an emotional concept—a higher level of probabilistic belief than a domain-specific one (Parr et al., 2022, p.217). Then, with the development of language, in a specific environment, the baby learns to associate the verbal label “hunger” with this concept—we can consider this verbal label to be a part of “semantic knowledge” about emotions. To summarize an emotional concept is a likely multi-modal semantic memory, which is not identical to semantic knowledge about emotions.

Adolphs (2017) showed that an emotional concept can be reliably dissociated from an emotional state based on the studies of the patient SM who had bilateral lesions to her amygdala. The patient’s emotional concept and semantic knowledge of fear were intact, but she did not show any signs of experiencing fear.

It seems useful to attempt the translations of Adolphs’ terms to the terminology used by other theorists. Panksepp and Biven (2012) refer to the “tertiary processes,” which are “emotional thoughts and deliberations (p.14).” They add:

Multiple emotional streams may cross in the thinking mind, creating an enormous variety of higher emotions that are often the focus of psychologists—pride, shame, confidence, guilt, jealousy, trust, disgust,^14^ dominance, and so forth with hundreds of possible variants. (p.15)

Paksepp and Biven thus acknowledge an increase in variability when moving from primary to tertiary level. We think that DISGUST, no less than Panksepp’s seven emotional systems, is a multi-tiered hierarchy. The first, instinctual level is described in Toronchuk and Ellis (2007a). At the secondary level, we can consider possible learning facilitated by aIC, BLA and their interconnections in acquiring knowledge of what is “clean/safe” and what is “dirty/pathogenic.” Maladaptive learning at that level, may contribute to clinical syndromes, such as contamination obsessions and washing compulsions based on misidentifications of the safe stimuli as noxious and based on a failure to update the inaccurate beliefs despite the data arriving from in the environment. At the tertiary level, we can consider psychological extensions of disgust described earlier, often culturally influenced, where stimuli or activities that are not inherently related to the protection of the body from pathogens are described as “dirty,” or “clean,” such as “unclean thoughts.”

Solms, following Freud’s definition, defined affect as the subjective aspect of the drive (Solms, 2021). Our reading of Solms’ terminology suggests that his definition of affect is analogous to what Adolphs calls “emotional experience” or “feelings.” Due to the subjective nature of affects, they are accessible only to the person experiencing them, unlike emotional expressions, including facial expressions, which can be observed by others.^15^

## How does this terminology apply to psychotherapy?

Behavioral psychotherapists, when working with OCD or phobias typically target interventions at the level of emotional expressions in Adolphs’ terms, such as the ritual prevention component in Exposure and Ritual Prevention (EX-RP) therapy. The exposure to a threatening or disgusting stimulus or context is a behavioral intervention in and of itself, where the patient’s behavior is the starting point. However, in the process of such exposure, the patient experiences feelings and is also likely exposed to the activation of emotional concepts. With that, phobic memories are unlikely to be extinguished in the process of exposure; what may happen instead is the formation of a new memory, which associates a previously threatening stimulus or context with a more benign reaction.

In psychodynamic psychotherapy, when the patient describes his experiences to the therapist, he shares data at the level of recollections of his past experiences, which he reflected on, classified, and labeled to the best of his abilities. There may be an explanatory gap in the patient’s narrative, where he reports suffering from discomfort that he is unable to name or describe. However, the level of verbal exchanges between the patient and the therapist is the level of semantic knowledge about emotions, not the level of functional biological states in Adolphs’ terms. Additionally, the patient may cry in a session, which, according to Adolphs, is an expression of an emotion.

The therapist may feel something in a session, which is the therapist’s affect. In psychodynamic psychotherapy, the therapist may choose to (a) classify her affective state verbally as, say, a “wish to care for the patient” (b) infer that this is countertransference, implying that this particular affect is related to the work in the therapeutic dyad (c) further infer that such countertransference is data in support of the patient either wishing to be cared for (complimentary countertransference), or of his wishing to care for others (concordant countertransference). The therapist may then incorporate her countertransferential impressions with other data into a preliminary psychodynamic formulation.

Panksepp and Biven (2012) acknowledged that psychotherapists interact with the patient at the tertiary level, not at the level of primary emotions (p. 25). Indeed, the patient does not talk to us, nor does he express his emotional states at the Panksepp’s primary level.^16^ The data we collect in psychodynamic therapy is what has been outlined above—the patient’s semantic knowledge, his expressions of emotions, his recollections, the therapist’s affects, and inferences about them. It is unlikely that we collect data directly at the level of the patient’s primary, instinctual emotional systems.

Therefore, should the therapist choose to attempt to classify the patient’s emotional distress by inferring which patient’s primary emotional systems are dysregulated, he would be making an inference two levels down in Panksepp’s hierarchy from the data collected. One possible example of such an inference seems to be made in the paper by Jackson and Solms (2013). They stated that separation distress/PANIC system was strongly implicated in OCD.

## Psychopathology and treatment for OCD, hypochondriasis, and health anxiety^17^

Athey et al. (2015) conducted a longitudinal study of 134 adult patients with severe OCD who received intensive residential treatment. The authors showed that at admission the patient’s disgust propensity (a person’s likelihood to experience disgust) was significantly associated with contamination/washing symptoms, but not hoarding, symmetry, or checking symptoms. Further, they have shown that at discharge disgust propensity improved and a reduction of disgust propensity was significantly associated with improvements in contamination/washing symptoms, but not symptoms in other domains. On this basis, the authors concluded that changes in disgust propensity can be considered predictive of changes in contamination/washing symptoms of OCD.

Knowles et al. (2016) conducted a similar study among 472 adolescents, 243 of whom had severe OCD and underwent residential treatment. In addition, the authors administered disgust proneness measures to adolescents with a primary mood disorder or a primary anxiety disorder diagnosis and not primary OCD. The results showed that a reduction in disgust proneness during treatment was significantly associated with reductions in multiple symptoms and that the strongest correlations were between reductions in disgust proneness and OCD symptoms. They did not infer a one-way causal relationship between disgust proneness and OCD but hypothesized about a reciprocal relationship between the two concepts.

In a pediatric study of 111 children diagnosed with OCD, Cervin and Perrin (2021) have shown that high levels of disgust and incompleteness measured at baseline predicted poorer treatment outcomes of behavioral therapy. They clarified that the felt sense of incompleteness was a feeling that things were “just not right” (p. 54). They assessed the three dimensions—fear, disgust, and incompleteness with Swedish versions of children’s Obsessive-Compulsive Core Dimensions Interview (OC-CDI) as part of the Dimensional Yale-Brown Obsessive-Compulsive Scale (DY-BOCS) interview with an item of disgust added to fear and incompleteness. The degree of fear at baseline was not predictive of the outcome. The study included a 13-month follow-up, but it was not controlled.^18^ Should their results be confirmed in a replicated and controlled study, Cerbin and Perry suggested that future treatment modifications that would target incompleteness and disgust could improve outcomes for at least a subset of children suffering from OCD.

Davey and Bond (2006) investigated a relationship between disgust, hypochondriasis, and health anxiety in adult patients. They have found highly significant correlations between trait disgust and disgust sensitivity and hypochondriasis and health anxiety; these correlations remained significant when trait anxiety was controlled for, which confirmed the results reported in an earlier study by Thorpe et al. (2003). Trait disgust and disgust sensitivity were measured with disgust propensity and sensitivity scales (DPSS); disgust sensitivity was additionally assessed with disgust sensitivity questionnaire (DSQ).

## Summary

The boundaries we draw between various bodily systems could be driven at times by the necessity to limit model complexity more so than by the data we collect. As an example, Ciaunica et al. (2023b) showed how truly complex and intertwined were the immune systems of the baby and the mother in pregnancy. While in a single human body the immune, endocrine, and central nervous systems are already tightly interrelated, in pregnancy there is an even more complex, delicate, and finely tuned balance of the mother’s systems, fetus’ systems, and the placenta. It could be that a functional task of protecting the internal milieu of a single organism or the nested system of several organisms, is the primary entity to consider, while a collection of various systems and tools of protection can change and evolve adaptively.

With these caveats in mind, our paper suggests that considering DISGUST as a primary emotional system likely brings theoretical and clinical benefits, including psychotherapeutic work with OCD, health anxiety, and other conditions where DISGUST plays a significant role. Our paper suggests that DISGUST is a complex and multilayered emotional system. We added data to Toronchuk and Ellis (2007a,b) work in support of DISGUST being a full-featured primary emotional system in Panksepp’s (1998) taxonomy, on par with SEEKING, LUST, CARE, PANIC, FEAR, RAGE, and PLAY.

In addition, we provided data in support of DISGUST acting as an affective partner of the immune system where both systems often work in concert on the same functional goal of protecting the internal milieu of the organism. Only by a comprehensive approach to all aspects of DISGUST can we gain a deeper understanding of the functioning of this system and further integrate this knowledge into clinical practice. It is also important to recognize that this system can be regulated by both external factors (such as learning, culture, and traumatic experiences) and internal physiological factors (such as hormones and immunity).

As noted in the section “Introduction,” we see this paper as a roadmap, which can help facilitate further research, theoretical developments, and clinical models. Specifically, we need: (a) more experimental data on DISGUST with rodents, as the experimental paradigm of observable retching-like behaviors was introduced only recently by Xie et al. (2022); (b) more evidence to clarify the possible role of the basal ganglia’s involvement in DISGUST; (c) more experimental data on the lateralization of functions in the insula and the basal ganglia; (d) more data to clarify the influence of the interoceptive and taste pathway differences between rodents and primates on the applications of rodent experiments to primates; (e) more data on the possible uniquely human re-representations in the insula suggested by Craig (2002).

We are hopeful that clinical models and their applications in psychotherapy and pharmacological therapy can consider the significant role of DISGUST in psychopathology. As applied to psychodynamic psychotherapy, we hope that our paper and the continued research on DISGUST would allow the psychodynamic theorists and clinicians to revise their theories of mental distress and therapeutic approaches. For some psychodynamic theorists, such as Nancy McWilliams, who studied with Silvan Tomkins, DISGUST has never lost its priority. However, in the neuropsychoanalysis community, where the clinical models are built on the foundation of Panksepp’s Affective Neuroscience—DISGUST is rarely if ever discussed in clinical case conferences, trainings, and lectures. We hope that our paper along with Toronchuk and Ellis’s work can help correct this omission.

## Data Availability

The original contributions presented in the study are included in the article/supplementary material, further inquiries can be directed to the corresponding author.

## Footnotes

^1^Sometimes, this condition is referred to as “Post-traumatic OCD.”

^2^Capitalized terms will be used in this paper to in accordance with Panksepp (1998) notation for the primary emotional systems.

^3^To add to this list, the disgust-evoking words associated with olfaction can include “stinky, putrid, foul, funky;” closely related gustatory associations can include “rancid, rotten;” visual associations can include “hideous, grisly.” Some of these words can be associated with multiple senses, e.g., “rotten” can be visual, olfactory or both.

^4^Which is something Panksepp (2007) himself acknowledged.

^5^DISGUST can also be conceptualized as participating in the allostatic mechanisms of the body. An example can include the upregulated DISGUST accompanying a period of partially downregulated immune system in pregnancy. DISGUST can also be conceptualized as participating in the allostatic mechanisms of the body. An example can include the upregulated DISGUST accompanying a period of partially downregulated immune system in pregnancy. This is an anticipatory process, where the upregulation of DISGUST is a measure that helps to maintain the protection of the internal milieu by proactively compensating for the expected deficit in the immune mechanisms of protection. Please see the DISGUST and the Immune System section for more details on this process.

^6^We will use terms “basic emotion” and “primary emotional system” interchangeably in this paper.

^7^The consequences of the failure of the immune system to identify the cancer cells as NonSelf components can be perceived as emotionally disgusting, e.g., the pictures of cancer on the cigarette packets.

^8^The researchers in the field of moral cognition and moral emotions (e.g., Moll et al., 2005) have expressed a slightly different viewpoint. They suggested that disgust could be a prototypic emotion, that has multiple domains, one of which could serve as a foundation for the development of complex moral feelings, e.g., the state of moral indignation. They have postulated that “condemnatory” emotions such as anger, contempt, disgust, and indignation were linked to aggression, and their experimental induction primarily involved the amygdala, septal area, hypothalamus, and cingulate cortex. Brain activity during “pure disgust” (devoid of moral undertones) or indignation was recorded while reading statements such as the following: “When walking down the street, you see a cat eating its own excrement” (Pure Disgust). “When you get home, you notice that the nanny has put a spider on your child’s face” (Indignation). Although disgust and indignation can be seen by some theorists as emotions that belong to the same family, Moll et al. (2005) showed that concomitant brain activity presented notable differences. Disgust was associated with activation in the amygdala, hippocampus, thalamus, anterior insula, anterior cingulate cortex, basal ganglia, and the orbitofrontal and temporal cortices. In contrast, indignation correlated with activity in the lateral division of the orbitofrontal cortex, anterior prefrontal cortex, insula, anterior cingulate cortex, right inferior frontal gyrus, right inferior temporal gyrus, and the anterior superior frontal gyrus.

^9^Please note that having some connections between the NTS and the amygdala via relays in the thalamus or the pons is not contradictory to these data. It is critical to look at the specific functional pathways – interoceptive and gustatory pathways. These inputs to the amygdala come from the insula in primates.

^10^A system, which is increasingly tied to issues of embodied cognition (Ciaunica et al., 2023a; Halley, 1991).

^11^The term “behavioral” is unfortunate in our opinion and likely implies “psychological.” Using adjective “emotional” instead of “behavioral” could be a better fit.

^12^There are some interesting parallels between pregnancy induced immunotolerance and psychopathology that can be linked to the notion of sensory attenuation. In brief, the sensory attenuation is the ability of a system to ignore certain inputs in order to act. Failures of sensory attenuation have been used in attempts to model some psychiatric conditions, including the OCD. For example, if a person failed to attenuate interoceptive evidence of physiological arousal, she may engage in behaviors that reduce the arousal persistently, because she cannot ignore the fact that she is still in a state of physiological arousal. The link with pregnancy here is the notion that the partial, functional downregulation of the mother’s immune response corresponds to the sensory attenuation at an immunological level, allowing the mother to bear a child (Bhat et al., 2021).

^13^In addition to the abovementioned data on the complex relationship between the DISGUST and the immune system it is worth mentioning the literature on the OCD subtype, which has been conceptualized as being caused by autoimmune conditions (Endres et al., 2022). Endres and colleagues used the Pediatric Autoimmune Neuropsychiatric Disorder Associated with Streptococcal infection (PANDAS) and Pediatric Acute onset Neuropsychiatric Syndrome (PANS) to establish the criteria for what they referred to as “autoimmune OCD” subtype.

^14^Please note that “disgust” they refer to here is the tertiary form of disgust, not the primary sensory emotion of disgust in Panksepp’s classification system.

^15^In addition to the terminology listed in this section, we can add a comment about the distinction between an emotional concept and an emotional feeling/affect *per se*. This can be described in Active Inference framework (Parr et al., 2022) as an emotional inference. In this framework, an emotion as a (propositional) concept is the representation of the state of Self that best explains both the interoceptive and exteroceptive evidence, where the interoceptive evidence could include the status of the immune system or the neurohormonal systems mediating DISGUST at lower scales. One interesting aspect of this view is that some individuals may have a diminished ability to recognize their emotional states (e.g., in alexithymia); these circumstances may be an appropriate fit for the therapeutic revision via mentalizing the individual’s physiology or homeostasis (Ainley et al., 2016; Fotopoulou and Tsakiris, 2017).

^16^A more precise statement would be that we hear narrative or see behavior, or experience feelings; and then we form a probabilistic belief (inference) about which Panksepp’s level these phenomena belong to.

^17^These studies are reviewed in addition to the clinical literature already reported by Toronchuk and Ellis (2007a,b).

^18^Childhood-onset OCD is thought to have a higher level of heritability than adult-onset OCD (see Bloch and Pittenger, 2010 for a comprehensive review).
